# Network topology and parameter estimation: from experimental design methods to gene regulatory network kinetics using a community based approach

**DOI:** 10.1186/1752-0509-8-13

**Published:** 2014-02-07

**Authors:** Pablo Meyer, Thomas Cokelaer, Deepak Chandran, Kyung Hyuk Kim, Po-Ru Loh, George Tucker, Mark Lipson, Bonnie Berger, Clemens Kreutz, Andreas Raue, Bernhard Steiert, Jens Timmer, Erhan Bilal, DREAM 6&7 Parameter Estimation consortium, Herbert M Sauro, Gustavo Stolovitzky, Julio Saez-Rodriguez

**Affiliations:** 1 T.J. Watson Research Center, Yorktown Heights, New York, USA; 2 European Molecular Biology Laboratory, European Bioinformatics Institute, Cambridge, UK; 3 Autodesk Research, San Francisco, CA, USA; 4 Department of Bioengineering, University of Washington, William H. Foege Building, Box 355061, Seattle, WA 98195-5061, USA; 5 Department of Mathematics, MIT, Cambridge, Massachusetts, USA; 6 BIOSS Centre for Biological Signalling Studies, University of Freiburg, Schänzlestr. 18, 79104 Freiburg, Germany; 7 Merrimack Pharmaceuticals One Kendall Square, Suite B7201, Cambridge, MA 02139, USA; 8 Physics Department, University of Freiburg, Hermann-Herder Str.3, 79104 Freiburg, Germany

## Abstract

**Background:**

Accurate estimation of parameters of biochemical models is required to characterize the dynamics of molecular processes. This problem is intimately linked to identifying the most informative experiments for accomplishing such tasks. While significant progress has been made, effective experimental strategies for parameter identification and for distinguishing among alternative network topologies remain unclear. We approached these questions in an unbiased manner using a unique community-based approach in the context of the DREAM initiative (Dialogue for Reverse Engineering Assessment of Methods). We created an *in silico* test framework under which participants could probe a network with hidden parameters by requesting a range of experimental assays; results of these experiments were simulated according to a model of network dynamics only partially revealed to participants.

**Results:**

We proposed two challenges; in the first, participants were given the topology and underlying biochemical structure of a 9-gene regulatory network and were asked to determine its parameter values. In the second challenge, participants were given an incomplete topology with 11 genes and asked to find three missing links in the model. In both challenges, a budget was provided to buy experimental data generated *in silico* with the model and mimicking the features of different common experimental techniques, such as microarrays and fluorescence microscopy. Data could be bought at any stage, allowing participants to implement an iterative loop of experiments and computation.

**Conclusions:**

A total of 19 teams participated in this competition. The results suggest that the combination of state-of-the-art parameter estimation and a varied set of experimental methods using a few datasets, mostly fluorescence imaging data, can accurately determine parameters of biochemical models of gene regulation. However, the task is considerably more difficult if the gene network topology is not completely defined, as in challenge 2. Importantly, we found that aggregating independent parameter predictions and network topology across submissions creates a solution that can be better than the one from the best-performing submission.

## Background

Predictive and mechanistic models are powerful tools to understand biological processes at the core of systems biology. Building models requires a list of molecular components and their interactions. This list can be assembled from prior knowledge and/or inferred, or reverse engineered, from dedicated experimental data [1-3]. This can be done using a simple causal formalism or, if enough mechanistic detail is available, by writing down the corresponding biochemical reactions. In both cases, once a reasonably well-characterized set of components and interactions is determined, these can be converted into a mathematical model. A common and natural way to model biochemical reactions is to derive a dynamical system, typically in the form of ordinary differential equations. These equations include associated parameters that quantify the underlying physicochemical processes such as protein binding and enzyme activity. The value of these parameters is often not available or even measurable, and needs to be estimated from experimental data [4-6]. An accurate estimation of the parameters is fundamental to quantitatively understand a system and provide reliable predictions [7,8].

In a real-life scenario of limited resources, the key question is how to design experiments that are most useful for parameter characterization [9], a decision process involving many variables. This problem falls in the category of budgeted learning formalized in the field of machine learning [10]. The first question raised is related to the experimental conditions that should be considered. When possible, data is collected upon application of perturbations to the network such as stimulation with extracellular ligands, chemical inhibition or gene over-expression. Moreover, data can be collected at different times after perturbation to provide information on the temporal evolution of the system. It is hence necessary to determine the identity and number of perturbations and whether to generate data from individual or combined perturbations. The next decision is related to the choice among a battery of technologies available to perform the measurements. These normally entail a trade-off between coverage, cost, and precision. For example, one can track over time the levels of a few proteins in single cells using GFP (Green Fluorescence Protein) tags and movies [11-18], or measure thousands of proteins in a few time-points with mass spectrometry [19]. How to choose among all these options is not obvious and, despite the critical importance of these questions, the problem of parameter estimation and iterative experimental design remains one of the hardest challenges in systems biology [4-6,9,20,21].

To explore this fundamental problem in a rational and unbiased fashion, we first set up the *parameter estimation challenge*, where we tried to reproduce the common setting in which an experimental laboratory uses instruments, expertise and an allocated budget (e.g. from a grant) to apply various experimental techniques to investigate a biological model system. To mimic this scenario, we built the model of a regulatory network for 9 genes based on differential equations describing the underlying molecular biology, including transcription and translation. We chose a model configuration that can generate non-trivial dynamic behavior. We then generated data with this model that included experimental noise and asked participants to find the model’s parameters. Each participant was given a budget of ‘credits’ that could be used to buy different experiments that reflected trade-offs between coverage, cost and resolution. We provided participants only the model structure and challenged them to estimate the hidden parameter values. Given that the true values of the hidden parameters were known, we could precisely assess the performance of the methods used by the 12 different teams that participated in the challenge. Remarkably, despite the complexity of the network and the limited data resources, some teams obtained highly accurate parameter values.

Besides the question of the algorithmic/experimental strategy used to infer the kinetic parameters of a model, we also addressed how well new connections in a network could be inferred. This is also a relevant question, as many canonical pathways are only approximations to the system under study. We therefore ran a second challenge, the *network topology inference challenge*, where participants were given an incomplete topology with 11 genes and asked to find 3 missing links in the model. This challenge was only partially solved, suggesting that inferring topology is a much harder challenge than parameter estimation. Finally, we observed that aggregating the participants’ parameter predictions and network topology submissions provided potentially better solutions than individual participants.

We complemented the analysis of the submissions by analyzing the participants’ algorithmic strategies and credit usage for data acquisition. We concluded that using fluorescent data from protein time courses is a key component of parameter estimation strategies, and that in both challenges aggregation created solutions that fared as well or better than the best performing approaches. We chose an *in silico* challenge framework in order to have a well-defined gold standard for evaluating submissions, but we believe the setup of this work emulates the experimental design choices faced by real laboratories, and thus the insights gained here provide insights for real experimental design when trying to determine the parameters of a gene regulatory network.

## Results

In both the *network topology* and *parameter inference* challenges, participants were asked to develop and/or apply optimization methods, including the selection of the most informative *in silico* experiments, to accurately estimate parameters and predict outcomes of perturbations from a model of a gene regulatory network. This challenge was divided in two parts. The first is *parameter inferenc*e, which was similar to the p*arameter estimation* challenge proposed in DREAM6, as explained here below. The second challenge is *network topology* and was unique to DREAM7.

### A realistic model of a gene regulatory network

In model 1 for the *parameter inference* challenge, participants were provided with the complete structure of the model (including expressions for the kinetic rate laws) for a gene regulatory network composed of 9 genes and modeled with differential equations. For each gene, both protein and mRNA are explicitly modeled and therefore the model contains 18 continuous variables. The complete model is available in the Additional file 1 (see *Model & Submissions*).

**Figure 1:**
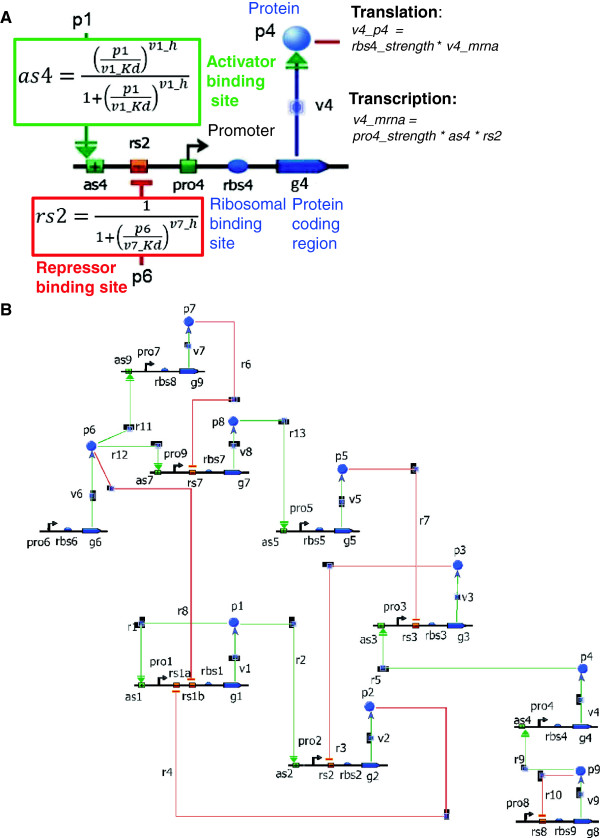
**Model and gene regulatory network of the parameter estimation challenge. A**. Example of a case of regulation of the transcription of coding sequence *g4* by proteins p1 and *p4*, respectively activator and repressor, through the activator (*as4, green box*) and repressor (*rs2*, red box) sites. The rate of production of *g4* is given by the transcription dependent on the promoter *pro4*. The rate of production of *p4* is given by the translation dependent on the ribosomal binding site *rbs4.***B**. Gene network from model 1 of the Parameter Prediction challenge consisting of 9 genes whose 45 parameters and the prediction of response to perturbations were requested from challenge participants.

The regulation of each gene was inspired from prokaryotes and modeled as follows: each gene can have, upstream of the protein coding region, an activator binding site, an inhibitory binding site, a promoter, and a ribosomal binding site (Figure 1A shows an example). Transcription rates were considered to be non-linear Hill-type functions of the regulatory inputs – activatory or inhibitory. A basal constant rate of transcription is assumed when a gene has no regulatory input. The transcription rate for a given gene is proportional to the promoter strength of the corresponding promoter and the translation rate is proportional to the ribosomal binding site’s strength. We assumed that transcription factors bind to operator sites independently and this is reflected in the transcription rate being expressed in a multiplicative form (e.g. as4 * rs2 in Figure 1A).

**Figure 2:**
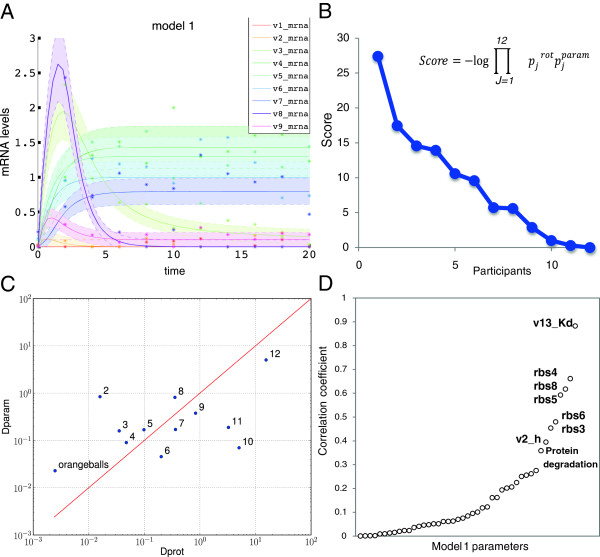
**Scores and correlation between parameter and protein prediction distances for model 1. A**. Graph representing the dynamics of the mRNAs from the 9 genes for model 1 network. Dots are the data with noise, lines represent the data without noise and shades the associated noise model **B**. Overall scores from the participants calculated from the p-values as indicated by the formula. P-values were obtained from the two different metrics used for challenge scoring described in Additional file 3: Figure S1. **C**. The participant distances defined for scoring the submitted predictions for the parameters and the protein perturbation predictions are plotted respectively in the y-axis *Dparam* and x-axis *Dprot*. Each team is represented by its rank number in the final scoring except for the best performer *Orangeballs.* The R^2^ coefficient for a linear fit in log-scale is 0.23; the red line is a visual reference for a perfect fit. **D**. For each of the 45 parameters in the model, the vector of parameter values submitted by the 12 participants is correlated (R^2^) to the unique vector of *Dprot* values, the protein perturbation prediction distance values. The graph shows the parameters ordered by increasing correlation value, with from left to right, pro5_strength, v10_Kd, pro3_strength, v9_Kd, v4_h, v8_Kd, v8_h, v1_Kd, v11_h, v1_h, pro7_strength, v4_Kd, v12_Kd, pro8_strength, rbs9_strength, v10_h, pro2_strength, v9_h, pro1_strength, v12_h, v5_h, pro4_strength, v3_h, v7_h, rbs7_strength, v3_Kd, rbs2_strength, pro9_strength, v6_h, rbs1_strength, v7_Kd, pro6_strength, v6_Kd, v11_Kd, v2_Kd, v5_Kd, v13_h, p_degradation_rate, v2_h, rbs3_strength, rbs6_strength, rbs5_strength, rbs8_strength, rbs4_strength, v13_Kd.

For each regulatory process, activation or repression, two parameters have to be estimated: the dissociation constant K_d_ and the Hill coefficient h. In model 1, for each protein production process, there are two parameters to be estimated: the promoter strength and the ribosomal binding site strength (see Figure 1A). The unit of time is normalized with the inverse of the mRNA degradation rate, and therefore time is non-dimensional: it is measured in units of the mRNA half-life (see Figure 2A for mRNA dynamics). All mRNA and protein degradation rates are constants in model 1 with a fixed value of 1 for mRNA, which is known by the participants. The protein degradation value is unknown and must be estimated from the data as part of the challenge. Therefore the total number of parameters to estimate in model 1 is 45: 18 from the regulation of 9 proteins (9 promoter and 9 ribosomal binding strength parameters), 26 from 13 regulation processes (13 K_d_, and 13 Hill parameters; see Table 1) and the protein degradation rate. The complete network is depicted in Figure 1B. The participants were required to provide the values for the 45 parameters of model 1 as well as the time courses of proteins *p*_3_, *p*_5_, and *p*_8_ under perturbed conditions defined below.

**Table 1:** Model parameters summary

**Parameter**	**Model 1**	**Model 2**
Promoter strength	9	**X**
rbs strength	9	**X**
Protien synthesis	**X**	16
Basals	**X**	2
Degradation rate	1	11
kd	13	16
Hill coefficient	13	16
Total	45	61

Parameters involved in the *parameter estimation challenge* and the *network topology challenge*. The nature of each parameter is indicated on the first column, and the number of parameters in Model 1 for the *parameter estimation* challenge and Model 2 for the *network topology challenge* are listed in the second and the third column, respectively.

Although the basic structure for both challenges is similar, the *network topology* challenge, referred to as model 2 hereafter, was simplified as compared to model 1 from the *parameter inference* challenge. In model 2, an incomplete structure of the regulatory interaction network topology was provided, with 3 missing regulatory links (see Additional file 2: Figure S2A). The gene regulatory network was composed of 11 genes where transcription was ignored and therefore only proteins were explicitly modeled (11 relevant variables). In contrast to model 1, the values of all protein degradation rate constants are not identical. In model 2, for each protein production process only the promoter strength has to be estimated and the protein production rate for a given gene is assumed to be proportional to the promoter strength of the corresponding promoter. Therefore, given that there are 16 regulatory interactions among the genes, the total number of parameters to estimate is 61: 3 for each regulatory interaction (16 synthesis rates, 16 K_d_, and 16 Hill parameters), 11 degradation rates, and 2 basal transcriptional rates for genes 5 and 11 that are not regulated by any other gene (see Table 1). Finally, participants were asked to provide the three missing links in the gene network (*r9*, *r10* and *r12* in Additional file 2: Figure S2A), as well as their associated parameters (K_d_ and h).

### A credit system mimicking a limited experimental budget

The participants are given a virtual budget of ‘credits’ to buy data from experiments (produced *in silico*). These experiments are used to estimate parameters of the gene regulatory networks in order to predict the dynamics under a perturbed condition in model 1 or to determine the missing links in model 2. The models were initially sketched out using TinkerCell [22] and simulated and tuned using Jarnac [23]. Perturbations that provided the data sets used for the parameter estimation were generated using Jarnac. We ran a first parameter estimation challenge (very similar problem to model 1) in DREAM6 (2011) with 3 models of 6, 7 and 9 genes. In DREAM6 we found compatibility issues when using different solvers. Therefore, in DREAM7 we ran these simulations with COPASI [24], Jarnac, Matlab [http://www.mathworks.com], SBML simulator [http://www.ra.cs.uni-tuebingen.de/software/SBMLsimulator/index.html] and Roadrunner [https://code.google.com/p/roadrunnerlib/] to ensure that different tools, which potentially have different underlying integrator routines, would provide the same results. The available perturbations and their associated costs are:

i. gene deletion, that produces the elimination of both mRNA and protein for the gene for 800 credits;

ii. siRNA-mediated knockdown, that increases the mRNA degradation rate 10-fold for 350 credits;

iii. a decrease of RBS (ribosomal binding site) activity that leads to a 10-fold decrease in translation rate for 450 credits.

Upon each of these types of perturbation, the teams could purchase data collected with different technologies, reflecting the relative ease or difficulty of acquiring this type of data in reality. Specifically, participants could buy time course data for:

i. protein abundance for 2 proteins of their choice at the highest resolution (every time unit) using fluorescence protein fusion for 400 credits;

ii. mRNA (for all genes) measured with a microarray, at either low resolution (every 4 time units) or high resolution (every 2 time units), at 500 and 1000 credits, respectively. Microarrays were only available in challenge 1, since the model of challenge 2 does not include mRNA;

iii. protein abundance for all proteins measured via mass spectrometry, also at high and low resolution for 500 and 1000 credits, respectively. This was available only in challenge 2, as an alternative to the microarrays of challenge 1.

Specific parameter values, namely the binding affinity (*K*_*d*_) and Hill coefficient (*h*), obtained from a gel-shift experiment, were also available for 1600 credits for a given transcription factor.

Finally, in both data modalities a noisy measurement is simulated by adding some noise to the deterministic value of each variable. More precisely, if *v* is the simulated value, we report as the measured value: v_noisy_ = v + 0.1 × g1 + 0.2 × g2 × v, where g1 and g2 are Gaussian random variables with standard deviations of 1. That is, for small v the standard deviation of v_noisy_ is close to 0.1, while when v is large, v_noisy_ amounts to measuring v with a standard error close to 20% of the true value. Note that if the value after noise addition is smaller than 0, the value of v_noisy_ is clipped at 0.

### Challenge results

The *network topology and parameter inference* challenge is composed of two parts corresponding to the two sub-challenges. The scoring of participants’ submissions reflects this two-tiered structure and is composed of two different scores (see *Methods* for a detailed description). The first score determines the ranking of teams in the *parameter inference challenge* by combining (i) the distance between the simulated and predicted protein concentration values and (ii) the distance between estimated and known parameters (model 1). The second score ranks the *network topology* challenge submissions based on the predictions for 3 missing links in the regulatory gene network (model 2).

In order to solve the challenge, participants were allowed to spend credits to procure data generated *in silico*. One could have designed a multi-optimization task where participants would have to balance their performance with budget expense. However, there is no standard or obvious way of deciding the optimal balance between these two terms. Thus, reflecting the common situation of an experimental laboratory that has been awarded a research grant with a budget for experiments to be spent in a certain amount of time, scoring in this challenge considered only their predictions. It did not take into account the amount of credits spent, and participants were encouraged to spend the whole budget.

As the questions posed in models 1 and 2 are different, identifying topology in one case and identifying parameters in the other, we decided to separate the two challenges and select a winner for each one. Figure 2B and Table 2 indicate that team *orangeballs* is the clear winner of the *parameter estimation* challenge with an overall score of 27.40 (see Additional file 3: Figure S1 for p-values). Table 3 shows that the winner of the *network inference* challenge is team *crux* with an overall score of 1.83 (see Additional file 3: Figure S2B for p-values).

**Table 2:** Scores and features of parameter inference challenge

**Model 1**	**Parameter distance D**^**param**^	**P-value for parameter predictions**	**Protein distance D**^**prot**^	**P-value for protein time course predictions**	**Score**	**Bayesian**	**Decompose network"**	**Selection of data**	**Sampling**
Orangeballs	0.0229	3.25E-03	0.002438361	1.21E - 25	27.4	no	yes	Game Tree	Sequential local search
2	0.8404	1.00E + 00	0.016023721	3.39E-18	17.5	no	no	Manual based on parameter uncertainty	Global method
3	0.1592	6.00E-01	0.035404398	4.45E-15	14.6	yes	no	Manual	LH
4	0.0899	1.88E-01	0.047495432	6.28E-14	13.9	no	yes	Manual	LM + Particle Swarm
5	0.1683	6.45E-01	0.09791128	4.01E-11	10.6	yes	no	Train + Sim	UKF
6	0.0453	1.37E-02	0.198785197	1.93E-08	9.6	no	no	A=Criterion	Local (LM)
7	0.1702	6.45E-01	0.362463945	2.90E-06	5.7	no	yes	Sensitivity analysis	Hybrid (Local + Global)
8	0.8128	1.00E + 00	0.356429217	2.53E-06	5.6	yes	no	Estimation of improved uncertainty	Global (MH)
9	0.3766	9.99E-01	0.817972877	1.34E-03	2.9	yes	yes	MI	ABC-SMC
10	0.0699	9.83E-02	19.32326868	1.00E + 00	1.0	no	yes	Minimize variance based on FI	Multistart local search
11	0.1883	7.29E-01	3.222767988	6.90E-01	0.3	no	no	Train + Sim	LH + DE
12	5.0278	1.00E + 00	14.77443631	1.00E + 00	0.0	no	no	Manual	Local method

Table for Model 1 of the *parameter inference challenge* contains anonymized teams (except for best performer) ordered by Score rank. Next to each team is listed its parameter distance and associated p-value, protein distance and associated p-value and the score. The last four columns indicate the features of the fitting strategies used by the participants. Abbreviations used for the features: *ABC-SMC*, Approximate Bayesian Computation with Sequential Monte Carlo; *DE*, Differential Evolution; *FI*, Fisher Information; *LH*, Latin Hypercube; *LM,* Levenberg-Marquardt; *MH*, Metropolis Hastings; *MI*, Maximize Mutual Information between parameters and output of experiments; *Train + Sim*, iterative steps of training on data and simulation to find most informative experiments; *Rank* rank experiments in top 10% of the A-Criterion (trace of the covariance matrix) according to price; *UKF*, Unscented Kalman Filtering.

**Table 3:** Scores and features of network topology challenge

**Model 2**	**Network score**	**p-value**	**Score**	**Link addition**
crux	12	1.49E-02	1.83	Manual
2	9	5.60E-02	1.25	Manual
3	8	1.07E-01	0.97	Manual first + algorithm
4	8	1.07E-01	0.97	Manual('logic reasoning')
5	8	1.07E-01	0.97	Manual
6	7	2.10E-01	0.68	Algorithm(Grenits)
7	6	3.83E-01	0.42	Manual
8	5	6.01E-01	0.22	Manual
9	4	8.01E-01	0.10	Did not participate
10	4	8.01E-01	0.10	Did not participate
11	3	9.86E-01	0.01	Manual
12	2	1.00E + 00	0	Algorithm GP-DREAM

Table for Model 2 of the Network topology Challenge contains anonymized teams (except for best performer) ordered by Score rank. Next to each team is listed their network score *s*^*network*^, associated p-value and the final score *Score*. The last column indicates the features of the link addition strategies used by the participants.

### Parameter inference results

An intriguing result of the *parameter inference* challenge is that although the best performing team *orangeballs* achieved the least error in both submitted parameters and protein predictions, these two metrics did not always correlate (see Table 2). The 10^th^ overall ranked team was second in parameter estimation but last in protein prediction. Conversely, the second overall ranked team was next to last in parameter estimation but second in protein prediction (Figure 2C). Although, as indicated by an R^2^ = 0.23 for the correlation of parameter distance *D*_1_^*param*^ to protein prediction distance *D*_1_^*prot*^ (Figure 2C), it is expected that some parameters do not influence the outcome of certain proteins, the discrepancy for the 2^nd^ and 10^th^ overall ranked teams was puzzling. After contacting the 10^th^ team we learned that their optimization objective was centered on the parameters and not on protein prediction. This underscores how the choice of scoring metric is not a trivial question and can dramatically influence the results [5]. Conversely, the 2^nd^ ranked team focused on the prediction of the protein values, and grouped together parameters that they found to be non-identifiable. Combinations of such non-identifiable parameters, such as *K*_*d*_ and *h* for a regulation reaction, were the quantities important to be able to correctly predict perturbed values for *p3, p5* and *p8*; thus, parameters far from the gold standard would still lead to good predictions of protein perturbation, as long as the implied combined quantities were close to the model solution. It is possible that different parameter values could lead to similar dynamical behavior and, as the 2^nd^ ranked team did, reproducing the original dynamical system behaviors might be more relevant than parameter estimation.

To further investigate this possibility, we analyzed the dependence of the protein perturbation predictions on each individual parameter, and calculated for each one of the 45 parameters the correlation of the vector of participants’ submitted parameter values to their protein prediction distance, *D*_1_^*prot*^. *D*_1_^*prot*^ was most dependent on the values of parameters directly involved in *p3, p5* and *p8* production such as, K_d_ for r13 (R^2^ = 0.88), rbs4 (R^2^ = 0 .66), rbs8 (R^2^ = 0.61), rbs5 (R^2^ = 0.59), rbs3 (R^2^ = 0 .45) (Figure 2D). Only protein degradation (R^2^ = 0 .35) is a global parameter. The strong dependency of *p3, p5, p8* prediction levels on only a few parameters may explain the low correlation between *D*_1_^*prot*^ and *D*_1_^*param*^.

### Aggregation of participants’ results

**Figure 3:**
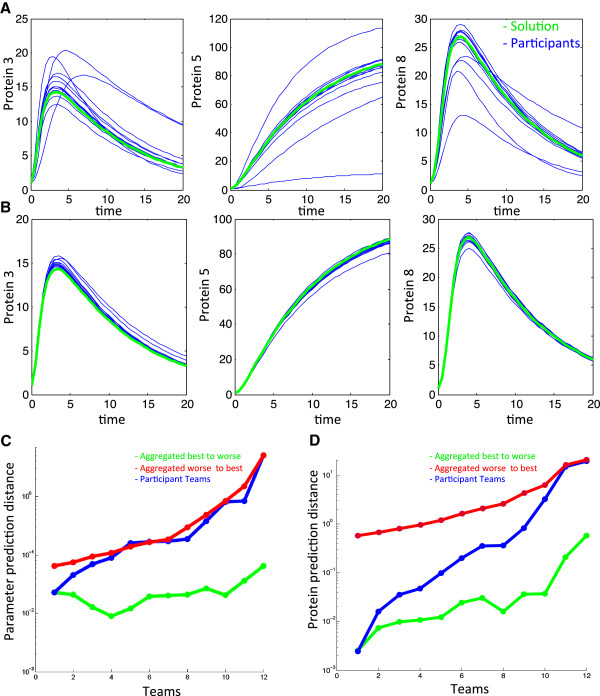
**Scores of aggregated participant results. A**. Protein concentrations of participants’ predictions (in blue) and the solution (green) are plotted against time for proteins *p3, p5* and *p8* under the perturbed conditions considered for scoring. **B**. Participant submissions are aggregated by averaging each protein concentration for individual time points, starting from the 2 best performing teams until all 12 teams are included. Each aggregated result is plotted in blue and the solution is plotted in green. **C**. Log scale distance to the solution of parameter predictions is plotted for participant teams ordered by rank (blue line) and geometric means of parameter predictions from teams ordered by number of aggregated teams following parameter distance rank (green line) or inverse rank order (red line). **D**. Log-scale distance to the solution of proteins *p3, p5* and *p8* under perturbed conditions is plotted for participant teams ordered by rank (blue line) and aggregated teams. Aggregations were computed for the predictions of the teams, ordered by number of aggregated teams ranging from 1 to 12, following prediction distance rank (green line) or inverse order (red line).

For model 1, most participants’ time-course predictions of proteins *p3, p5* and *p8* are close to the solution (Figure 3A blue lines) but, as seen in other DREAM challenges [25,26], aggregated participant submissions are robust, as the prediction is close to the gold standard and ‘buffers’ outliers (Figure 3B blue lines). Predictions were aggregated by averaging each protein concentration for individual time-points starting from the best performing team, followed by averaging the first and second best performing teams, and so on until all 12 teams were included.

This phenomenon also occurs when aggregating the participants’ submitted parameters by geometric mean using the same procedure as above. *D*_1_^*param*^ for this aggregation by geometric mean shows that for up to eight aggregated teams, the aggregated team submission is closer to the solution when compared to *D*_1_^*param*^ for the best individual team submission (Figure 3C blue line and green line). However, performance of teams aggregated from worst to best (that is, the worst performing team, followed by the worst and second worst performing teams and so on until all 12 teams are included) fares overall poorer than individual teams (Figure 3C red line). In a real situation, without the gold standard, one would not know which participants fare better; in such a case performance of randomly aggregated predictions would fall between these two extreme cases of aggregation. Importantly, aggregating parameters of all teams fares as well as the third best performing team, and therefore it is a better strategy to aggregate results from multiple teams than choose a single given method.

Results are mitigated when one considers *D*_1_^*prot*^ as a measure of the effectiveness of the aggregation of solutions. Indeed, choosing as a solution the aggregation of all teams brings a *D*_1_^*prot*^ that is worse than eight of the teams (Figure 3C blue line and last point in green line). This is due to the fact that participants obtained very good predictions for the protein measurements: the winner *orangeballs* obtained a relative p-value of 1.21. 10^-25^, compared to 3.35. 10^-3^ for parameter estimation results (see Additional file 3: Figure S1). In practical terms, the aggregated prediction of all teams as shown by Figure 3B is still a very good prediction for the perturbations effect.

**Figure 4:**
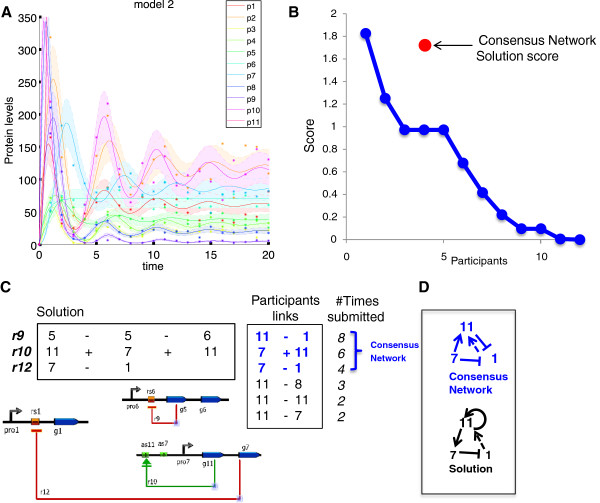
**Dynamics and scores of the network topology challenge. A**. Time courses of the proteins from the 11 proteins in the model 2 network. Dots are the data with noise, lines represent the data without noise and shades the associated noise model. **B**. Ordered scores from the participants as well as the score of the consensus solution defined as the 3 most submitted links. Scores were calculated from the p-values as indicated in Methods, Additional file 2: Figure S2 and Additional file 1. **C**. The 3 links *r9, r10, r12* composing the solution to the Network Inference challenge are shown in their numeric (top left) and diagram (bottom left) notations. The list of submitted participant links is shown (right) in its numeric notation as well as the number of times such links were submitted. The links colored in blue indicate the consensus network composed of the 3 most submitted links whose score is indicated in (B). **D**. Diagrams of consensus network of links (blue) and solution (black). Dashed arrow indicates an indirect regulation.

In model 2, to find the participants’ consensus 3 missing links, we counted how often links were submitted by participants and chose the 3 most popular ones (Figure 4C). Although this strategy limits the possibility of obtaining higher scores, as most participants submitted links that controlled only one gene, the score of this consensus submission matches the best performing team *crux* (Figure 4B & C).

### Analysis of participants’ strategies and experimental credit usage

**Figure 5:**
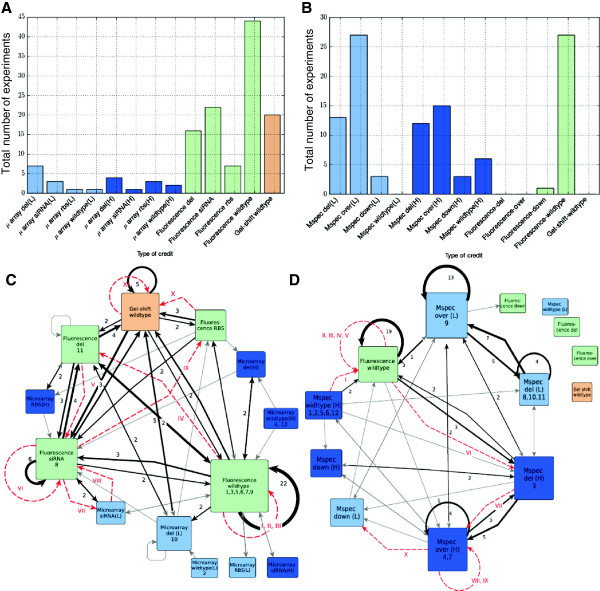
**Analysis of experimental credit usage in challenges A.** Histogram indicating the number of times credits were spent on an experiment for the *parameter estimation challenge*. The nature of the experiments is indicated on the horizontal axis. **B**. Histogram indicating the number of times credits were spent on an experiment for the *network topology challenge*. **C**. Diagram indicating the sequence of experiments performed in the p*arameter estimation challenge*. Each box represents a different experiment and the arrows indicate the sequence followed. Dark arrows represent the most used paths with numbers indicating usage, and grey arrows indicate a single usage. The path of the winning team is shown with red arrows and the order of the experiments is indicated via roman numerals. **D**. Diagram indicating the sequence of experiments performed in the *network topology challenge*. Each box represents a different experiment and the arrows indicate the sequence followed as in (C).

The various types of data and perturbations were used differently by teams for each of the challenges. Available data types were slightly different between the challenges, since mass spectrometry data was not available for challenge 1 and microarray data was not available for challenge 2. Of the 13 different possible combinations of experiments, the most demanded one was the measurement via fluorescent microscopy of time-courses of two proteins (Figure 5A). This experiment, which was also the cheapest one, was requested in 68% of all the teams’ inquiries and also for the best performing team *orangeballs*. The most demanded type within the fluorescent experiments was wildtype (33%). The low-resolution wildtype microarray was the least demanded, as it was given as initial data to the participants. In 15% of the solution strategies, teams used credits to purchase the Michaelis constant and Hill coefficient parameters via gel-shift experiments.

For the *network inference* challenge, credits were mostly spent on mass-spectrometry, although fluorescent microscopy of wildtype time-courses came in a strong second (see Figure 5C). In contrast to the *parameter estimation* challenge, no team directly bought parameters via a gel-shift experiment. Alternative strategies can also be seen on the paths followed by the participants when purchasing experimental data (Figure 5B and D for models 1 and 2, respectively). In brief, winning strategies for model 1 acquired microarray data to have precise measurements on genes and then mainly used fluorescent time-course experiments to refine parameter values. For model 2, wild-type fluorescence data was used to cheaply find disagreements between data and model and then mass spectroscopy experiments with perturbations were used to test for potential missing links.

These differences indicate alternative strategies for the solution of both challenges (see details in Tables 2 and 3). Briefly, 5 out of 12 teams used a Bayesian framework, and 4 used some strategy based on decomposing the network into smaller sub-networks for further analysis. The sampling of the parameter space was performed with a variety of methods: local, often using multi-start strategies to avoid getting stuck in local minima; global; or hybrid. The key question for model 1 was how to choose new informative experiments. To address it, most teams used *in silico* perturbations to infer which experiments would be more informative. They defined this using different metrics, such as Fisher information, mutual information, etc. Particularly innovative was the strategy of the winning team *orangeballs* based on a game tree, as it could easily be adapted to bigger networks. For challenge 2, asking which experiment was the most informative had to be combined with a strategy to explore the network topology to find missing links. Few teams used algorithms for network inference, while most teams, including the winner, used heuristics based on manual inspection of the network and intuition. As an illustration of the different approaches, the best performers for each challenge describe in detail their strategies in the following sections.

### Winning strategy for the *parameter estimation* challenge (from team *orangeballs*)

The basic idea of our approach was to compute a maximum-likelihood fit of the model parameters given observed data purchased from *in silico* experiments. Computing the likelihood function is straightforward because once the model parameters have been specified, we have all the equations needed to simulate time courses and calculate likelihoods based on the specified noise model. Choosing an optimal sequence of data purchases is challenging, however: because of the limited budget, it is critical to select experiments most likely to be informative even when the model behavior is initially largely unknown.

We began our analysis of each model by buying time courses of all proteins under wildtype conditions. These experiments were by far the cheapest and allowed us to start making initial guesses at parameter values. For example, the protein degradation rate can be estimated from the time course of a non-regulated protein (e.g., *p6* in Figure 1B), the RBS values can be read off from steady-state values of [protein]/[mRNA], and in cases where we have a guess that regulatory coefficient values are close to 1 (as they often turned out to be), promoter strength values can be found from steady-state mRNA concentrations. Observation of the dynamics of protein and mRNA time courses also sometimes allows estimation of dissociation constants.

Having initial guesses of the parameters, we then viewed the problem of choosing successive data purchases as a game tree of possible sequences of experiments, with the goal being to identify paths most likely to reduce the uncertainty as much as possible at minimum cost. Given that the optimal sequences change as data is purchased (revealing information about the model parameters), we generally tried to find experiments to perform early on that (i) were likely to be necessary regardless of the actual parameter values, or (ii) would provide information distinguishing the most disparate possibilities (e.g., in some cases it was impossible to tell initially whether a regulator was performing full activation or zero activation).

Because of the combinatorial complexity of possible data purchase paths, however, it was critical to apply heuristics to estimate the utility of purchases and to limit the search space. Given the heuristic nature of the search and the relatively small size of the networks, we found it most practical to map out plausible purchase paths on paper rather than codifying our game tree search scheme. We now describe a few key heuristics we developed that we found most valuable.

• Steady-state values provide the cleanest measurements of parameters because having a multiplicity of measurements of the same steady-state value allows for averaging out noise. Moreover, combining different steady-state values enables direct inference of activation and repression parameters (*k*_*d*_ and *h* coefficients). Indeed, at steady-state, the following relations hold:

$$\begin{array}{l} \left(\text{mRNA degradation rate}\right)*\left[\text{mRNA}\right]\\ \;=\left(\text{pro strength}\right)*\left(\text{regulatory terms}\right) \end{array}$$

and

$$\left(\mathrm{protein degradation}\right)*\left[\text{protein}\right]=\left(\text{rbs strength}\right)*\left[\text{mRNA}\right]$$

• Combining these equations,

$$\begin{array}{l} \left(\text{regulatory terms}\right)\\ \;=\left(\text{mRNA degradation rate}\right)*(\text{protein degradation rate})\\ \;*[\text{protein}]/((\text{pro strength})*(\text{rbs strength})) \end{array}$$

• Considering for the moment the case of a single repressor, there are two unknowns, *K*_*d*_ and *h*, and the left side has the form

$$\frac{1}{1+{\left(\frac{\left[\text{regulatory protein}\right]}{K_{d}}\right)}^{h}}$$

• Different steady-states under experimental perturbations yield values of the right side corresponding to different values of the regulatory protein concentration, and taking ratios of these values isolates the effect of the regulation. It follows that 3 steady-state measurements are theoretically enough to determine *K*_*d*_ and *h*. In light of noise, however, it is very important that the steady states cover a range of concentrations of the regulatory protein that includes or comes near the value of *K*_*d*_.

• For the purpose of obtaining new steady-state measurements at minimal cost, a trade-off has to be considered between protein measurements (which get 2 new steady states) and mRNA measurements (which get values for all genes, but at much lower resolution). Additionally, a given perturbation typically only produces new steady states for a small number of genes because the effect of the perturbation is often mitigated downstream (by saturation of an activator or repressor). We found that 2-protein measurements generally seemed to be most cost-effective with a few exceptions.

• Most protein and mRNA time courses simply converge to steady-state behavior, but in cases with interesting dynamics, the time trace information is highly informative and can allow inference of parameters with fewer perturbations; this is important to keep in mind to reduce costs.

• For some regulations, the only option is to measure *K*_*d*_ and *h* directly using (expensive) gel-shift experiments. These problematic cases arise when it is difficult to keep the protein concentration at the scale of *K*_*d*_ for a reasonable amount of time; most often this happens when *K*_*d*_ is very small and the regulating protein increases quickly in concentration. Another case is if *K* is much larger than any observed concentrations of the regulatory protein.

These heuristics collectively allowed us to drastically limit the number of candidate experiments to consider at each purchasing step, typically just to one or two possible experiments directed at investigating each unknown parameter. Because the scoring function was based on total squared relative error, prioritizing the least constrained parameters was clearly advantageous and further reduced the search space. Additionally, whenever we were able to identify components of a model that functioned approximately independently, we applied a divide-and-conquer approach to analyze each component in isolation – again limiting the combinatorial explosion of search paths – and then aggregated the results,

As a final note, after finding potential perturbations to run using these heuristics, we were able to test whether the experiments were likely to achieve their objectives by simply simulating the effects of the perturbations and checking whether different values of the parameters led to noticeably different time traces. We found this simple check to be very useful in helping decide which data to buy.

### Winning strategy for the *network inference* challenge (from team *crux*)

From the point of view of statistical methodology, inferring missing links in a gene regulatory network model based on experimental data constitutes a model discrimination issue. We applied the classical maximum likelihood methods to address this benchmark challenge.

For the given error model which can be described by a probability distribution *ρ* and a presumed network structure *M*, the likelihood is a product

$$L\left(\theta \right)=\prod_{i}\rho \left(y_{i}\left|M,\theta \right)\right)$$

over all data points *y*_*i*_ interpreted as a function of the parameters *θ*. Evaluating the likelihood *L* requires the numerical integration of the ODEs which we performed using the CVODES algorithm of the SUNDIALS package [27]. Estimating the parameters by the maximum likelihood method requires numerical optimization of the likelihood. For this purpose, the trust-region method (MATLAB, R2011a, The Mathworks Inc., Natick, MA) was applied. Since gradient-based optimization critically relies on the accuracy of the first derivatives and finite difference approximations are known to be inappropriate for ODEs [28], the first derivatives were calculated by solving the so-called sensitivity equations [27]. The Hessian is approximated as a product of the Jacobian to obtain second derivative information [29].

Since the model is nonlinear with respect to the parameters, the likelihood landscape can exhibit local minima. Therefore, optimization was repeated using multiple initial guesses. For this purpose, we used Latin hypercube sampling to efficiently explore the parameter space [30].

To assess the model’s ability to explain the data, we used the least-squares goodness of fit statistic.

$$\chi^{2}=\sum_{i}\frac{{\left(y_{i}-x_{i}\right)}^{2}}{\sigma^{2}}$$

where x denotes the concentrations predicted by the model. Moreover, likelihood ratios have been utilized to statistically test whether extending the model by additional parameters significantly improves the fit. Since in the challenge the measurement errors were given as normally distributed, log-likelihood ratios are in fact proportional to differences of χ^2^.

The profile likelihood [31] was used to assess parameter identifiability. Informative experimental conditions were found by exploring the model predictions within the parameter confidence intervals, i.e. by simulating the model for all parameter vectors obtained within the profile likelihood calculation [31]. In general, perturbations and observations which are informative for estimating parameters are characterized by large variations of the model predictions which are reduced if the respective conditions are evaluated experimentally [32]. In the case of several potential model structures, this procedure can be repeated for each model to identify experimental setups where candidate models yield qualitatively different predictions.

Initially, we performed less costly protein measurements for the wildtype setting to have a minimal amount of experimental information enabling the application of the tools introduced above. In this stage, we already gained confidence that the data required an extension of the model allowing for oscillations. Introducing a negative feedback on protein p1 mostly improved our outcome.

In the next stage, we favored mass spectrometry experiments since they provide comprehensive information of all regulators and targets. Having a complete data set for a perturbation setting is advantageous to minimize the risk of erroneously proposing links. Moreover, we preferred high-resolution data to obtain as much information as possible about the dynamics. We noticed that missing links with a Hill-type kinetic are only identifiable if the concentrations of the regulator cover the range around the respective Michaelis constant *K*_*d*_. Therefore, we primarily concentrated on perturbations where we expected largely different concentration ranges of potential regulators.

Additional file 4: Table S1 provides a summary of our iterative experimental planning decisions. We could correctly identify the regulatory effects of *p7* and *p11*, we found *p1* as negatively and *p11* as positively regulated targets and could thereby reach 12 points in the assessment discussed in Section 2.3.1. We could not find the link from *p5* on the common promoter of the genes of *p5* and *p6*. However, after the organizers provided the true parameters to the participants, we recognized that this link is difficult to detect due to the fact that for almost all perturbations the concentration of *p5* is clearly above the Michaelis constant *K*_*d*_ = 17.9 of this missing link.

## Discussion

In order to evaluate how well mechanistic models could be built upon inferred biological networks, we tested the accuracy of model parameter predictions and missing link identification. Surprisingly, with a limited amount of data, participants were able to reliably predict the value of the parameters and temporal evolution of 3 proteins under perturbed conditions in the *parameter inference challenge*. Participants did not fare so well in the *network topology challenge*; although 2 of the 3 links involved were identified (Figure 4), none of the teams found more than one correct link.

### Aggregation of participant results

DREAM results for a diverse set of challenges have recurrently demonstrated the “wisdom of crowds” phenomenon, where aggregation of participants’ results has proven to give robust and top performing results [3,25,26]. The *network topology* and *parameter estimation* challenge is very different in nature from other DREAM challenges, not only because it is the first one to address the dynamics of a bio-molecular network using a given biochemical (mechanistic) model, but also because it uses a credit system for participants to obtain *in silico* experimental data in an iterative manner.

In spite of these original features, we have been able to obtain, as in other DREAM challenges, a robust and high-performing set of predictions based on the geometric mean for the parameters and arithmetic mean for the protein predictions (Figure 3C, D). Geometric mean proved an adequate approach to address the issue that parameter values predicted by different teams could vary several orders of magnitude. Notably, this aggregation method resulted in several solutions with a reduced distance to the parameter values (Figure 3D). It is not clear whether the success of aggregating results is partially due to more data sets being used, since each participant had access to potentially different experimental data. Note, however, that this is not equivalent to a single participant using the combination of the data used by all participants.

For the *network topology challenge*, although only *crux* had statistically significant results, the consensus 3 missing links obtained by majority voting to select the most submitted links had a top performing score (Figure 4B, C). Interestingly, only one of the three consensus links is correct (*r12* Figure 4B), but the two others correctly implicate genes 1, 7 and 11, although the direction and nature of the regulatory link is incorrect. This proves how difficult it is to differentiate between regulatory diagrams based solely on limited experimental data and perturbations (Figure 4D).

### Participants’ methods and credit usage

The strategies for data acquisition were different for the *parameter inference* and the *network topology* challenges. As shown in the histograms of Figure 5, participants in the first challenge used most of their credits to collect fluorescent data from time-courses of two proteins. In the second challenge participants equally used mass-spectroscopy experiments and fluorescent protein time-courses. The interpretation of such diverging strategies can be illustrated from the sequence of data acquisitions of the best performing teams (Figure 5B, D). For the first challenge, *orangeballs* acquired microarray data to have precise measurements on genes and then mainly used fluorescent time-course-experiments to refine parameter values. On the second challenge, *crux* first used credits on wildtype fluorescence data, to cheaply obtain a setting with qualitative disagreement between data and model, and then used mass spectroscopy experiments with perturbations to test for potential missing links. Also, Table 2 suggests that best performing teams mostly took a manual approach for credit usage; automatic methods relying only on a numerical criterion seem not to perform as well for these mechanistic models.

## Conclusions

Our results show that from a defined gene network model it is possible to accurately determine the kinetic parameters of a gene regulatory circuit, given simple fluorescent-based experimental data and an adequate inference strategy. More generally, our results suggest that state-of-the-art parameter estimation and experimental design methods can in principle determine accurate parameters of biochemical models of gene regulation, but the task is considerably more difficult or maybe impossible to unequivocally solve if the knowledge of the topology is not precise, as often is the case.

As they stand, this study and the underlying data and models are a useful resource for those interested in developing parameter inference methods and to benchmark them against state-of-the-art methods. This strategy could be extended and tested on larger, genome size gene networks using whole-cell models [33], or alternatively, laboratory-produced data on synthetic circuits could be used instead of *in silico* data. Expanding these methods may allow precise determination of kinetic reaction parameters in cases where direct experimental measurements do not exist or are difficult to obtain.

## Methods

### Scoring the *parameter estimation* challenge

#### Distance between simulated and predicted values

For model 1, participants were requested to predict three protein time courses from *t*_0_ = 0 to *t* = 20 seconds with a sampling Δ*t = 0.5*, for a total of *N* = 41 data points. We denote *t*_*i*_ the time at data point *i*. The predicted and simulated levels of protein *k* are denoted *p*_*k*_^*pred*^ and *p*_*k*_^*sim*^(*t*) with *k* = 3, 5, 8 as the proteins required to be predicted are *p*_3,_*p*_5,_ and *p*_8_ (see Figure 1B). These predictions were required for an experiment where the network is perturbed simultaneously with a 10-fold decrease of *r*9_*kd*_, a 2-fold increase in rbs3 strength and a 10-fold increase of rbs5 strength. These proteins and perturbed states were chosen so that predictions could not be trivially inferred from purchased data.

Because the initial conditions are given, the real challenging predictions take place after some time has elapsed from *t*_0_. We considered that time to be 10 intervals of time and thus evaluated the predictions from the 11^th^ time point onwards. Accordingly, the squared distance between predicted and measured protein abundances for the model we used is:

$${D_{1}}^{\text{prot}}=\frac{1}{3\left(N-11\right)}\sum_{k=1}^{3}\sum_{i=11}^{N}\frac{{\left({p_{k}}^{\text{pred}}\left(t_{i}\right)-{p_{k}}^{\text{sim}}\left(t_{i}\right)\right)}^{2}}{\left(\sigma_{b}^{2}+\sigma_{s}^{2}{\left({p_{k}}^{\text{sim}}\left(t_{i}\right)\right)}^{2}\right)}$$

Note that the squared difference terms are normalized with the variance, and the variance follows the noise model that was implemented in the data provided (with σ_*b*_ = 0.1 and σ_*s*_*=* 0.2). The quantity $\sigma_{s}^{2}$ represents a baseline, signal-independent, measurement noise, and $\sigma_{s}^{2}$ represents a signal-dependent measurement noise.

Finally, the difference is divided by (3 * (*N*-11)) the number of terms being added, to obtain a mean distance value. The distance $D_{1}^{\text{prot}}$ was computed for each team.

To statistically evaluate the performance of the teams, a relative null hypothesis was created from this distance, based on the predictions of all the participants. For each protein, we chose at random one of the 12 participant’s predictions for the first time point *p*_*k*_^*pred*^(*t*_*i*_), then at random one of the 12 predictions for the next time point, and so on. We therefore obtained a value of $D_{1}^{\text{prot}}$ that would correspond to one possible random choice of predictions amongst all the participants. Repeating this process a large number of times, we generated a distribution of squared distances, from which a p-value can be estimated for $D_{1}^{\text{prot}}$. That p-value will be denoted as $p_{1}^{\text{prot}}$ (see Additional file 3: Figure S1A).

#### Distance between estimated and known parameters

As degradation rates are equal for all proteins, only one degradation parameter has to be determined and thus model 1 has *N*_*p*_ = 45 parameters to be considered for scoring.

Let us denote as *v*_*i*_^*pred*^ and *v*_*i*_^*real*^ the predicted and actual parameter values used in the simulations, where *i* runs between 1 and *N*_*p*_. The mismatch between estimated and true parameters will be assessed on the log-scale. In this way, a mismatch by a factor of x has the same penalty independent of the parameter’s nominal value and the ratio is also independent of physical unit changes. Therefore the “distance” between predicted and real parameters is calculated as follows:

$$D_{1}^{\text{param}}=\frac{1}{N_{p}}{\sum_{i=1}^{N_{p}}\left(\log\left(\frac{v_{i}^{\text{pred}}}{v_{i}^{\text{real}}}\right)\right)}^{2}$$

Similar to the case of the distance between simulated and predicted protein abundances, a relative null hypothesis is created from the distance between estimated and known parameters based on the predictions of all the participants. For each parameter, we chose at random one of the 12 participant’s predictions for the parameter. We therefore obtained a value of *D* that would correspond to one possible random choice of predictions amongst all the participants. By doing the same process a large number of times, we generated a distribution of distances between known and estimated parameters, from which a p-value can be estimated for *D*_1_^*param*^. That p-value will be denoted as *p*_1_^*param*^ (see Additional file 3: Figure S1B).

For each team the overall score Score_1_ combining both parameters and protein values is defined as

$$\text{Scor}e_{1}=-\log\left({p_{1}}^{\text{prot}}\cdot {p_{1}}^{\text{param}}\right)$$

### Scoring the *network topology* inference challenge

#### Distance between the estimated and true network

For model 2 we requested the prediction of 3 missing links of the network as shown in Additional file 2: Figure S2A. Protein dynamics are different from Model 1 and in particular include oscillatory behavior (Figure 4A). In order to facilitate the task of the participants, the possible universe of links was reduced by a rule stating that (i) genes could only establish a maximum of two regulating links and (ii) a link could regulate up to two genes in the same operon. Hence, six gene interactions had to be indicated by the participants composed of three links regulating up to two genes and also defining whether the gene regulation is activating (+) or repressing (−).

For each of the three predicted links i = 1,2,3, we defined a score *s*_*i*_^*link*^ that gives a value between 0 and 6 depending on how well the link is captured: a perfect prediction of the link is rewarded with 6 points, while correctly predicting only the starting gene, the end gene, or the sign of the effect, is given a lower score. Specifically, the score is computed as

$${s_{i}}^{\text{link}}=L_{i}+N_{i},$$

Where *L*_*i*_ = 6 if one connection has all its elements correctly predicted (that is, the source gene, the sign of the connection, and the destination gene are all correct). For the special case that a link regulates an operon composed of two genes and both connections are correct, reflecting the correct prediction of two connections, a doubled number of points *L*_*i*_ = 12 was awarded. Otherwise, *L*_*i*_ = 0 if some element of the connection is not fully correct. If *L*_*i*_ = 6 or 12 then *N*_*i*_ = 0 and the scoring for that link is complete, with a final score *s*_*i*_^*link*^ of 6 or 12, respectively. In case a link is not correctly predicted (*L*_*i*_ = 0), *N*_*i*_ adds to the score a value (less than 6) indicating how good the prediction is. Each gene interaction is positive or negative and composed of a source and a destination gene. Then, *N*_*i*_ is increased by 1 for each correctly predicted gene, and by 2 if the destination gene and the nature of the regulation (i.e. +/−) are correct. Correct (+/−) predictions without the correct associated genes are given no points. Some examples of these scores are provided in the non-exhaustive Additional file 5: Table S2.

The scores for the predictions of the three missing links are added in a global score

$$s^{\text{network}}={s_{1}}^{\text{link}}+{s_{2}}^{\text{link}}+{s_{3}}^{\text{link}}$$

A null model is calculated by generating a distribution of scores from a large number of surrogate gene networks obtained by randomly adding 3 links that follow the connection rules indicated in the challenge description. For each participant, a *p*_2_^*netw*^ p-value associated with the score under the null hypothesis is calculated (see Additional file 2: Figure S2B), and then the final score *Score*_2_ for this challenge is computed as

$$\text{Scor}e_{2}=-\text{log}\left({p_{2}}^{\text{netw}}\right)$$

## Dialogue for reverse engineering assessment and methods 6 (DREAM6) &7 parameter estimation consortium

We indicate D6 or D7 if teams participated only in DREAM6 or DREAM7

team ALF D6

Alberto de la Fuente, Andrea Pinna, Nicola Soranzo. CRS4 Bioinformatica c/o Parco Tecnologico della Sardegna, Edificio 3 Loc. Piscina Manna 09010 Pula ITALY

team amis2011

Adel Mezine : 1 Artemis Llamosi : 1 & 3 (current address : Université Paris Diderot, Sorbonne Paris Cité, MSC, UMR 7057 CNRS, 75013, Paris, France) Véronique Letort : 2 Arnaud Fouchet : 1 Michele Sebag : 3 Florence d’Alché-Buc : 1 & 3

1 : IBISC EA 4526, Université d’Evry Val d’Essonne, 23 Bd de France, 91000, Evry, France,

2 : Ecole Centrale Paris, Laboratory of Applied Mathematics and Systems (MAS), F92295 Châtenay Malabry, France,

3 : INRIA Saclay, LRI umr CNRS 8623, Université Paris Sud, Orsay, France.

team BadgerNets D6

Devesh Bhimsaria, Parameswaran Ramanathan, Aseem Ansari, Parmesh Ramanathan

Dept. of Electrical & Computer Engineering Tel: (608) 2630557 University of Wisconsin, Madison Fax: (608) 2621267 Madison, WI 537061691

Team BIOMETRIS D7

Laura Astola, Jaap Molenaar, Maarten de Gee, Hans Stigter, Dijk van Aalt-Ja, Simon van Mourik, Johannes Kruisselbrink

Wageningen University Plant Sciences Subdivision Mathematical and Statistical Methods, PO box 100 6700 AC Wageningen, Netherlands

team BioProcessEngi D6

Julio Banga, Eva Balsa Canto, Alejandro F Villaverde, Oana Chis, y David Henriques.Bioprocess Engineering Group Institute for Marine Research (IIMCSIC), R/Eduardo Cabello, 6. Vigo 36208, Galiza, Spain

team COSBI D6

Paola Lecca

The Microsoft Research – University of Trento Centre for Computational and Systems Biology. Piazza Manifattura 138068 Rovereto, Italy

current affiliation is Centre for Integrative Biology University of Trento Via delle Regole, 101,38123 Mattarello (TN), Italy Email: paola.lecca@unitn.it

team Crux

Clemens Kreutz, Andreas Raue, Bernhard Steiert, Jens Timmer

Freiburg Institute for Advanced Studies (FRIAS), University of Freiburg, Albertstr. 19, 79104 Freiburg, Germany

Institute of Bioinformatics and Systems Biology, Helmholtz Center Munich, Ingolstaedter Landstr. 1, 85764 Neuherberg, Germany

Physics Department, University of Freiburg, Hermann Herder Str. 3, 79104 Freiburg, Germany

team ForeC_in_HS D7

Julian Brandl, Thomas Draebing, Priyata Kalra, Ching Chiek Koh, Jameson Poon, Dr. Sven Sahle, Dr. Frank Bergmann, Dr. Kathrin Huebner, Prof. Dr. Ursula Kummer. University of Heidelberg, Seminarstraße 2, 69117 Heidelberg, Germany

team GIANO6 D6

Gianna Toffolo, Federica Eduati and Barbara Di Camillo

University of Padova Department of Information Engineering Via Gradenigo 6B 35131 Padova, ITALY

team ipk_sys D6

Syed Murtuza Baker, Kai Schallau, Hart Poskar, Bjorn Junker, Swetlana Friedel. Data Inspection group and Systems Biology Group, Leibniz Institute of Plant Genetics and Crop Plant Research.

team KroneckerGen D6

David R Hagen [1,2] and Bruce Tidor [1-3] drhagen@mit.edu

1) Department of Biological Engineering, Massachusetts Institute of Technology, Cambridge, MA, USA

2) Computer Science and Artificial Intelligence Laboratory, Massachusetts Institute of Technology, Cambridge, MA, USA

3) Department of Computer Science and Electrical Engineering, Massachusetts Institute of Technology, Cambridge, MA, USA

team 2pac

Cihan Oguz, Tyson Lab,

Departments of Biological Sciences Virginia Polytechnic Institute & State University Blacksburg, VA 24061 USA

team LBM D6

Michael Mekkonen, MIT

Lu Chen, WUSTL School of Medicine

Vipul Periwal, LBM, NIDDK, NIH

team ntu D7

Ching Chang1, Juo Yu Lee1, MeiJu May Chen2, YuYu Lin3 and ChienYu Chen1,2

1 Department of BioIndustrial Mechatronics Engineering, National Taiwan University, Taipei, Taiwan;

2 Genome and Systems Biology Degree Program, National Taiwan University and Academia Sinica, Taipei, Taiwan;

3 Graduate Institute of Biomedical Electronics and Bioinformatics, National Taiwan University, Taipei, Taiwan

team orangeballs

Po-Ru Loh, George Tucker, Mark Lipson, Bonnie Berger

Department of Mathematics, MIT, Cambridge Massachusetts

team Reinhardt

Christian Lajaunie, Edouard Pauwels, Jean Philippe Vert

Centre for Computational Biology, Mines ParisTech, Fontainebleau, F77300 France Institut Curie, Paris, F75248, France U900, INSERM, Paris, F75248, France

team TBP D7

Orianne Mazemondet, Friedemann Uschner Katja Tummler, Max Floettmann, Sebastian Thieme, Abel Vertesy, Marvin Schultz, Till Scharp, Thomas Spiesser, Marcus Krantz, Ulrike Mänzner, Magdalena Rother, Matthias Reis, Katharina Albers, Wolfgang Giese and Edda Klipp from Theoretical Biophysics Humboldt Universität zu Berlin

team thetasigmabeta

Juliane Liepe, Siobhan MacMahon, Paul Kirk, Sarah Filippi, Christopher Barnes, Thomas Thorne, Michael P.H. Stumpf Centre for Integrative Systems Biology and Bioinformatics, Imperial College London London SW7 2AZ UK

team ZiBIOSS D6

Zhike Zi, BIOSS Centre for Biological Signalling Studies, University of Freiburg, Schaenzlestr. 18 s, 79104, Freiburg, Germany

## Competing interests

The authors declare that they have no competing interests.

## Authors’ contributions

DC, GS, HS, JRS, KHK, PM, TC designed the challenge, DC generated the data, TC EB PM did the scoring of the challenge, PM JRS TC wrote the manuscript. All authors read and approved the final manuscript.

## Supplementary Material

Additional file 1 **Supplementary material files– Models and Submissions – model and data for challenge are provided as supplementary material as well as participants’ submissions.** Models are provided in MATLAB and Systems Biology Markup Language (SBML format) and the submissions name reflects the rank except for the best performing teams. They are also available at the DREAM site (http://www.the-dream-project.org/challenges/network-topology-and-parameter-inference-challenge). Click here for file

Additional file 2: Figure S2 Network topology challenge gene network and scores A. Gene network for model 2 of 11 genes and 45 parameters where links *r9, r10, r12* were missing and whose identity challenge participants had to determine. B. A score is calculated based on the 3 different links predicted and a p-value is calculated based on the distribution of randomly generated links used as a null-hypothesis (see main text). Click here for file

Additional file 3: Figure S1 Score calculation of the Parameter Estimation Challenge. A. A distance as shown by the equation is calculated based on the 45 parameters predicted values and a p-value is calculated when compared to a distribution of randomly generated relative null-hypothesis. B. A distance as shown by the equation is calculated based on the predicted protein concentration value for *p3*, *p5* and *p8* and a p-value is calculated when compared to a distribution of randomly generated relative null-hypothesis. Click here for file

Additional file 4: Table S1 Summary of the experimental design considerations of team crux for the network inference challenge. The second column denotes the chosen experimental conditions in the notation used during the challenge. The arguments underlying their decisions are denoted by abbreviations. Wild-type measurements provide data for substantially fewer credits (argument “WT”). Such measurements have been chosen initially to obtain a setting with a reasonable set of identifiable parameters. Data with high resolution over time (argument “High-Res”) provides more detailed information about the dynamics and was therefore expected to be more efficient for distinguishing potentially missing links with similar qualitative effects. Using a measurement technique providing data for all compounds (argument “All”) is advantageous to obtain a comprehensive overview of the effect of a perturbation. The argument abbreviated by “Range” indicates the fact that missing links are only identifiable, if the concentration range of the regulator is not far from the respective Michaelis constant *K*_*d*_. Therefore we performed perturbations affecting the concentration range of potential regulators in a desired direction. Finally, we had to take into account the remaining credits indicated by the argument “Budget”. Click here for file

Additional file 5: Table S2 Table used to score the submitted links for network topology challenge A link is defined by a source and a destination gene, and a source gene may or may not have two destination genes. Each row on the table represents a possible link submission. *N*_*i*_ represents the number of points given for the submitted link, where *i* stands for incorrect and *c* a correct prediction of the source and destination gene. Note that correct (+/−) predictions without the correct gene give no points. Click here for file
