# Supplementary material for: Network topology and parameter estimation: from experimental design methods to gene regulatory network kinetics using a community based approach
Source: BMC Syst Biol. 2014 Feb 7;8:13. doi: 10.1186/1752-0509-8-13 (PMC3927870; doi:10.1186/1752-0509-8-13)
Supplement: Additional file 2: Figure S2 — Network topology challenge gene network and scores A. Gene network for model 2 of 11 genes and 45 parameters where links r9, r10, r12 were missing and whose identity challenge participants had to determine. B. A score is calculated based on the 3 different links predicted and a p-value is calculated based on the distribution of randomly generated links used as a null-hypothesis (see main text). [file 1752-0509-8-13-S2.pdf]

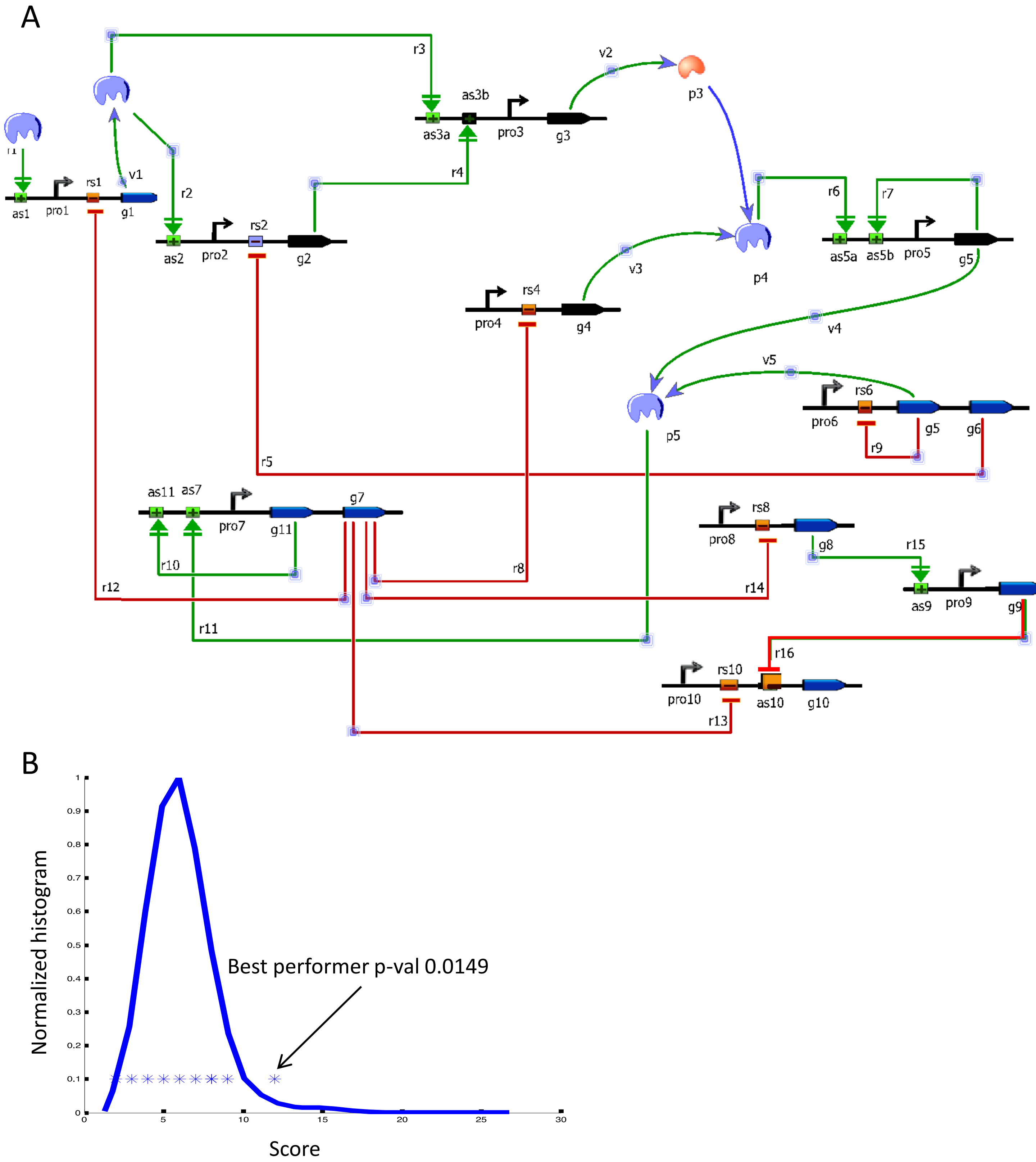

Figure S2. Network Topology Challenge gene network and scores A. Gene network for model 2 of 11 genes and 61 parameters where links r9,r10,12 were missing and whose identity challenge participants had to Determine.**B.** A score is calculated based on the 3 different links predicted and a p-value is calculated based on the distribution of randomly generated links used as a null-hypothesis.
