# Supplementary material for: Network topology and parameter estimation: from experimental design methods to gene regulatory network kinetics using a community based approach
Source: BMC Syst Biol. 2014 Feb 7;8:13. doi: 10.1186/1752-0509-8-13 (PMC3927870; doi:10.1186/1752-0509-8-13)
Supplement: Additional file 3: Figure S1 — Score calculation of the Parameter Estimation Challenge. A. A distance as shown by the equation is calculated based on the 45 parameters predicted values and a p-value is calculated when compared to a distribution of randomly generated relative null-hypothesis. B. A distance as shown by the equation is calculated based on the predicted protein concentration value for p3, p5 and p8 and a p-value is calculated when compared to a distribution of randomly generated relative null-hypothesis. [file 1752-0509-8-13-S3.pdf]

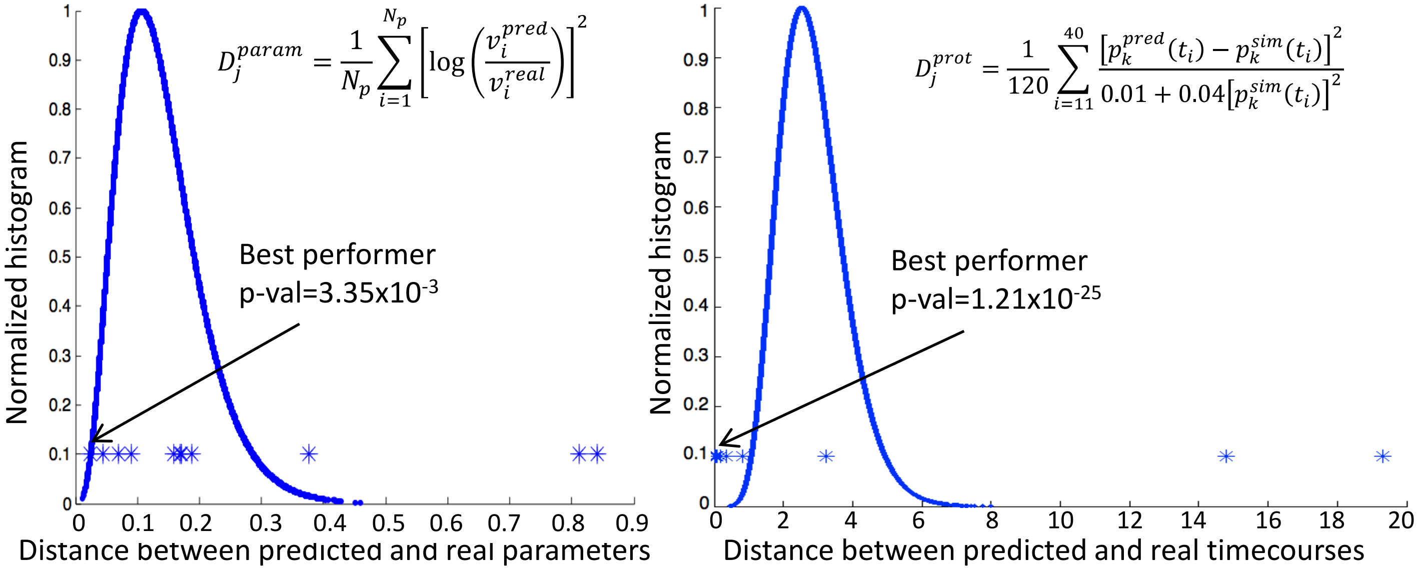

Figure S1. Score calculation of the Parameter Estimation Challenge

A. A distance as shown by the equation is calculated based on the 45 parameters predicted values and a p-value is calculated when compared to a distribution of randomly generated relative null-hypothesis. **B.** A distance as shown by the equation is calculated based on the predicted protein concentration value for p3, p5 and p8 and a p-value is calculated when compared to a distribution of randomly generated relative null-hypothesis.
