# Supplementary material for: Network topology and parameter estimation: from experimental design methods to gene regulatory network kinetics using a community based approach
Source: BMC Syst Biol. 2014 Feb 7;8:13. doi: 10.1186/1752-0509-8-13 (PMC3927870; doi:10.1186/1752-0509-8-13)
Supplement: Additional file 4: Table S1 — Summary of the experimental design considerations of team crux for the network inference challenge. The second column denotes the chosen experimental conditions in the notation used during the challenge. The arguments underlying their decisions are denoted by abbreviations. Wild-type measurements provide data for substantially fewer credits (argument “WT”). Such measurements have been chosen initially to obtain a setting with a reasonable set of identifiable parameters. Data with high resolution over time (argument “High-Res”) provides more detailed information about the dynamics and was therefore expected to be more efficient for distinguishing potentially missing links with similar qualitative effects. Using a measurement technique providing data for all compounds (argument “All”) is advantageous to obtain a comprehensive overview of the effect of a perturbation. The argument abbreviated by “Range” indicates the fact that missing links are only identifiable, if the concentration range of the regulator is not far from the respective Michaelis constant Kd. Therefore we performed perturbations affecting the concentration range of potential regulators in a desired direction. Finally, we had to take into account the remaining credits indicated by the argument “Budget”. [file 1752-0509-8-13-S4.pdf]

| Step | Purchased measurements | Perturbation type | Perturbation target | Experimental technique | Arguments | Remaining credits |
|---|---|---|---|---|---|---|
| 1 | array_mod_2_wildtype_high .tab | Wildtype | - | Mass spectrometry | WT | 9600 |
| 2 | p8_p9_mod_2_wildtype.tab | Wildtype | - | Fluorescence microscopy | WT, High-Res | 8600 |
| 3 | p3_p4_mod_2_wildtype.tab | Wildtype | - | Fluorescence microscopy | WT, High-Res | 8200 |
| 4 | p5_p7_mod:2_wildtype.tab | Wildtype | - | Fluorescence microscopy | WT, High-Res | 7800 |
| 5 | p1_p2_mod_2_wildtype.tab | Wildtype | - | Fluorescence microscopy | WT, High-Res | 7400 |
| 6 | p10_p11_mod_2_wildtype.t ab | Wildtype | - | Fluorescence microscopy | WT, High-Res, All | 7000 |
| 7 | array_mod_2_del_p7_high.t ab | Deletion | p7 | Mass spectrometry | Range, High-Res, All | 5200 |
| 8 | array_mod_2_over_p4_high .tab | Over-expression | p4 | Mass spectrometry | Range, High-Res, All | 3850 |
| 9 | array_mod_2_over_p8_high .tab | Over-expression | p8 | Mass spectrometry | Range, High-Res, All | 2500 |
| 10 | array_mod_2_over_p11_hig h.tab | Over-expression | p11 | Mass spectrometry | Range, High-Res, All | 1150 |
| 11 | array_mod_2_dwn_p7_low. tab | Knockdown | p7 | Mass spectrometry | Range, All, Budget | 250 |

**Table S1. Summary of the experimental design considerations of team crux for the network inference challenge.** The second column denotes the chosen experimental conditions in the notation used during the challenge. The arguments underlying our decisions are denoted by abbreviations. Wild-type measurements provide data for quite less credits (argument "WT"). Such measurements have been chosen initially to obtain a setting with a reasonable set of identifiable parameters. Data with high resolution over time (argument „High-Res") provides more detailed information about the dynamics and was therefore expected to be more efficient for distinguishing potentially missing links with similar qualitative effects. Using a measurement technique providing data for all compounds (argument „All") is advantegous to obtain a comprehensive overview of the effect of a perturbation. The argument abbreviated by „Range" indicates the fact that missing links are only identifiable, if the concentration range of the regulator is not far from the respective Michaelis constant $k_D$. Therefore, we performed perturbations affecting the concentration range of potential regulators in a desired direction. Finally, we had to account the remaining credits indicated by the argument „Budget".
