# Supplementary material for: Network topology and parameter estimation: from experimental design methods to gene regulatory network kinetics using a community based approach
Source: BMC Syst Biol. 2014 Feb 7;8:13. doi: 10.1186/1752-0509-8-13 (PMC3927870; doi:10.1186/1752-0509-8-13)
Supplement: Additional file 5: Table S2 — Table used to score the submitted links for network topology challenge A link is defined by a source and a destination gene, and a source gene may or may not have two destination genes. Each row on the table represents a possible link submission. Ni represents the number of points given for the submitted link, where i stands for incorrect and c a correct prediction of the source and destination gene. Note that correct (+/−) predictions without the correct gene give no points. [file 1752-0509-8-13-S5.pdf]

Source gene	(+/-)	Destination gene	(+/-)	Destination gene	Value of N_i
i	*i*	*i*	*i*	*i*	*0*
c	*i*	*i*	*i*	*i*	*1*
i	*c*	*i*	*i*	*i*	*0*
i	*i*	*c*	*i*	*i*	*1*
i	*i*	*i*	*c*	*i*	*0*
i	*i*	*i*	*i*	*c*	*1*
i	*i*	*i*	*c*	*c*	*2*
i	*c*	*c*	*i*	*i*	*2*
c	*i*	*c*	*i*	*i*	*2*
c	*i*	*i*	*i*	*c*	*2*
c	*i*	*c*	*i*	*c*	*3*
c	*i*	*i*	*c*	*c*	*3*
c	*c*	*c*	*i*	*i*	*3*
c	*c*	*c*	*i*	*c*	*4*
c	*i*	*c*	*c*	*c*	*4*

Table S2 Table used to score the submitted links for Network Topology challenge
A link is defined by a source and a destination gene, and a source gene may or may not have two destination genes. Each row on the table represents a possible link submission. N_i represents the number of points given for the submitted link, where *i* stands for incorrect and *c* a correct prediction of the source and destination gene. Note that correct (+/-) predictions without the correct gene give no points.
